# What Lies behind the Ischemic Stroke: Aortic Dissection?

**DOI:** 10.1155/2014/468295

**Published:** 2014-12-02

**Authors:** Turgut Deniz, Ersel Dag, Murat Tulmac, Burcu Azapoglu, Caglar Alp

**Affiliations:** ^1^Department of Emergency Medicine, Faculty of Medicine, Kirikkale University, 71350 Kirikkale, Turkey; ^2^Department of Neurology, Faculty of Medicine, Kirikkale University, 71350 Kirikkale, Turkey; ^3^Department of Cardiology, Faculty of Medicine, Kirikkale University, 71350 Kirikkale, Turkey

## Abstract

*Introduction*. Some cases with aortic dissection (AD) could present with various complaints other than pain, especially neurological and cardiovascular manifestations. AD involving the carotid arteries could be associated with many clinical presentations, ranging from stroke to nonspecific headache. *Case Report*. A 71-year-old woman was admitted to emergency department with vertigo which started within the previous one hour and progressed with deterioration of consciousness following speech disorder. On arrival, she was disoriented and uncooperative. Diffusion magnetic resonance imaging (MRI) of brain was consistent with acute ischemia in the cerebral hemisphere. Fibrinolytic treatment has been planned since symptoms started within two hours. Echocardiography has shown the dilatation of ascending aorta with a suspicion of flap. Computed tomography (CT) angiography has been applied and intimal flap has been detected which was consistent with aortic dissection, intramural hematoma of which was reaching from aortic arch to bilateral common carotid artery. Thereafter, treatment strategy has completely changed and surgical invention has been done. *Conclusion*. In patients who are admitted to the emergency department with the loss of consciousness and stroke, inadequacy of anamnesis and carotid artery involvement of aortic dissection should be kept in mind.

## 1. Introduction

Acute aortic dissection is one of the most dramatic cardiovascular emergencies unless promptly recognised and treated. Classical acute aortic dissection (AD) has been described as presenting with sudden, severe back or chest pain characterized as ripping or tearing in nature [1, 2]. However, not all ADs present with classic symptoms, and establishing the diagnosis can be difficult when the classic pattern of pain is absent [3]. Aortic dissection is not diagnosed on its initial presentation in 15–43% of cases [4, 5]. Many cases with AD were reported to present with various complaints other than pain, especially neurological and cardiovascular manifestations. Aortic dissection involving the carotid arteries is reported to be associated with many clinical presentations, ranging from stroke to nonspecific headache [1, 6, 7].

The most important point in acute ischemic stroke treatment is that it provides reperfusion with an early fibrinolytic treatment [8, 9]. If any contraindication defined in the guidelines does not exist intravenous fibrinolytic therapy should be started immediately in early hours of acute ischeamic stroke. One of the absolute contraindications in the guideline is aortic dissection. This case report is aimed at emphasizing the importance of ruling out diagnosis of aortic dissection before fibrinolytic treatment in acute ischemic stroke.

## 2. Case Report

**Figure 1:**
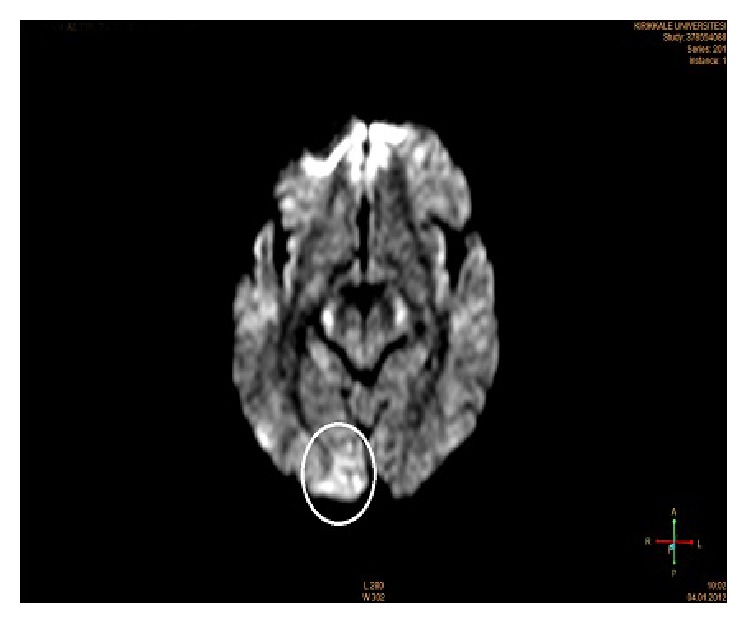
Axial diffusion-weighted echo-planar MR image (apparent diffusion coefficient shows an area of restricted diffusion in right occipital lobe).

**Figure 2:**
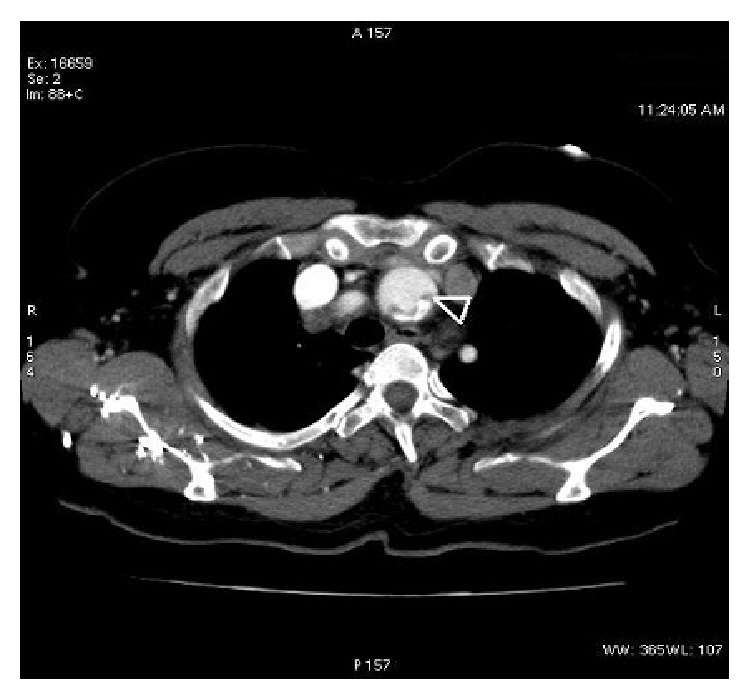
CT scan with contrast enhancement disclosed dissection in the brachiocephalic trunk (arrowhead).

In this case, a female patient, aged 71, with vertigo which started within the last one hour and progressed with weakness of arms and loss of the consciousness following speech disorder, has been brought to the emergency service. Her general condition was impaired; the vital status of the patient who was unconscious was evaluated (BP: 90/50 mmHg; pulse: 54 p/min; respiration: 18/min). Auscultation of heart revealed a regular rate and rhythm without any murmurs, and peripheral arterial pulses were symmetrical. Emergent chest X-ray and brain computed tomography were normal. Patient's diffusion MRI of brain was consistent with acute ischemia in right occipital lobe (Figure 1). Fibrinolytic therapy has been planned since symptoms started within two hours. Even though there was not any suspicion of aortic dissection in anamnesis and physical examination, bedside transthoracic echocardiography has been done. Echocardiography has shown significant aortic root dilatation and flap suspicion on a parasternal long axis view. Thereafter thorax CT angiography has been applied, and in the aortic root intimal flap has been detected which is consistent with aortic dissection which has been expanding from aortic arch to proximal bilateral carotid arteries (Figure 2). Based on these findings the treatment has been changed and surgical invention has been planned.

## 3. Discussion

AD is a dramatic medical emergency with a high mortality rate (1% to 2% per hour for 24 hours). AD is most commonly seen in hypertensive people between 50 and 70 years of age and occurs more often in men than in women. Primary neurological presentation is rare, and potentially lethal treatments like fibrinolysis may be initiated, especially in patients presenting with stroke and aphasia [10, 11]. In addition to migrating chest pain (85% of patients) and/or back pain (46%), additional signs such as pulse deficit (30%), hypotension (21%), pericardial effusion (29%), aortic regurgitation (30%), abnormal ECG (69%), and elevated D-dimer may point toward aortic dissection. Side branch involvement of the supra-aortic vessels with dissection of the common carotid or subclavian artery occurs in 15% to 41% of cases [12, 13]. Unrecognized patients with AD would be exposed to lethal complications of fibrinolytic therapy. Common delay in diagnosis of AD which results in higher mortality and narrow therapeutic time window of stroke reflects great diagnostic challenge to emergency clinicians [14].

In our patient risk factors for AD, hypertension and age, are also typically present in most stroke patients. Patient was uncooperative and anamnesis was inadequate. The mediastinum was not enlarged on portable chest X-ray. Additionally there was not any accompanying finding like pulse deficit or abnormal EKG. At first glance AD was not considered because of the lack of signs other than unexplained hypotension. Dissection-induced hypotension may have elicited cerebral perfusion insufficiency. ED physicians should consider screening bedside echocardiography in patients with unexplained hypotension.

Bedside echocardiography can be performed easily without time delay or transport of the patient, and we consider it a helpful complementary tool for the current diagnostic workup. Emergency physicians are capable of performing basic cardiac scans with focused training. The sonographic images presented are intended to immediately influence short-term management of possible aortic dissection in any patient entering the emergency room [13]. Even though there was not any suspicion of aortic dissection in anamnesis and physical examination, bedside echocardiography has been done. Echocardiography has shown the dilatation of ascending aorta and flap suspicion.

Stroke is one of the major causes of mortality worldwide. AD may present with predominant neurological symptoms of acute ischemic stroke (AIS) without the typical appearance of chest pain, hypotension, and absent peripheral pulses [11, 15, 16]. Guidelines and similar articles should warn colleagues not to administer fibrinolytics without careful evaluation of signs and symptoms of AD in patients with AIS. This advice was provided in the American Stroke Association Stroke Council's 2010 guidelines for the diagnosis and management of patients with thoracic aortic disease, as well as in the 2003 guidelines for early management of patients with AIS [17]. Intravenous thrombolysis is the only approved treatment for AIS within 4-5 hours from symptom onset. Systematic investigations of the underlying mechanism of cerebral ischemia have rarely been performed. Because of the narrow time window, the underlying stroke pathogenesis may not be investigated, and therefore careful selection of appropriate candidates may not be performed [18, 19]. Arterial dissection accounts for up to 20% of AISs [20]. Carotid artery dissection (CAD) has been associated with AD, reported in as many as 41% of AD cases [21]. Because of its association with aortic dissection, early recognition of CAD might affect the decision regarding thrombolysis for AIS [22].

Diffusion MRI imaging was performed after the brain CT of our patient and did not reveal any hemorrhagic focus. Upon suspicion of intimal flap during transthoracic echocardiography, CT angiography of aortic root revealed type A AD which involved proximal parts of common carotid arteries. Cerebral angiography is the gold standard for the diagnosis of CAD; however, this technique is invasive and is not readily available in many centers [22].

Intravenous fibrinolytic therapy for AIS is now generally accepted [23]. The US Food and Drug Administration (FDA) approved the use of intravenous rtPA in 1996, partly on the basis of the results of the National Institute of Neurological Disorders and Stroke rtPA Stroke Study, in which 624 patients with AIS were treated with placebo or rtPA within 3 hours of symptom onset, with approximately one half treated within 90 minutes [24]. However, more recent and globally crucial documents regarding stroke therapy do not mention thrombolysis as a medication error in AIS caused by AD [23, 25]. The same is true for recommendations for imaging in patients with AIS published in 2009 [26].

AIS secondary to unrecognized AD may lead to not only inappropriate thrombolysis, but also further worsening of the catastrophe. Colleagues should be repeatedly warned, especially in guidelines, to exclude this possibility quickly [17].

## 4. Conclusion

Bedside echocardiography has a great importance in stroke patients who have aortic dissection risk factors and who are planned to be treated by fibrinolysis treatment. AD, which is one of the reasons of AIS, is a diagnosis which should necessarily be excluded in order to apply fibrinolytic therapy. In patients who are admitted to the emergency department with the loss of consciousness and stroke, inadequacy of anamnesis and carotid artery involvement of aortic dissection should be kept in mind.
